# Upper Extremity Freezing and Dyscoordination in Parkinson's Disease: Effects of Amplitude and Cadence Manipulations

**DOI:** 10.1155/2013/595378

**Published:** 2013-08-21

**Authors:** April J. Williams, Daniel S. Peterson, Michele Ionno, Kristen A. Pickett, Gammon M. Earhart

**Affiliations:** ^1^Program in Physical Therapy, Washington University in St. Louis School of Medicine, St. Louis, MO 63108, USA; ^2^Department of Neurology, Washington University in St. Louis School of Medicine, St. Louis, MO 63110, USA; ^3^Department of Anatomy and Neurobiology, Washington University in St. Louis School of Medicine, St. Louis, MO 63110, USA

## Abstract

*Purpose*. Motor freezing, the inability to produce effective movement, is associated with decreasing amplitude, hastening of movement, and poor coordination. We investigated how manipulations of movement amplitude and cadence affect upper extremity (UE) coordination as measured by the phase coordination index (PCI)—only previously measured in gait—and freezing of the upper extremity (FO-UE) in people with Parkinson's disease (PD) who experience freezing of gait (PD + FOG), do not experience FOG (PD-FOG), and healthy controls. *Methods*. Twenty-seven participants with PD and 18 healthy older adults made alternating bimanual movements between targets under four conditions: Baseline; Fast; Small; SmallFast. Kinematic data were recorded and analyzed for PCI and FO-UE events. PCI and FO-UE were compared across groups and conditions. Correlations between UE PCI, gait PCI, FO-UE, and Freezing of Gait Questionnaire (FOG-Q) were determined. *Results*. PD + FOG had poorer coordination than healthy old during SmallFast. UE coordination correlated with number of FO-UE episodes in two conditions and FOG-Q score in one. No differences existed between PD−/+FOG in coordination or number of FO-UE episodes. *Conclusions*. Dyscoordination and FO-UE can be elicited by manipulating cadence and amplitude of an alternating bimanual task. It remains unclear whether FO-UE and FOG share common mechanisms.

## 1. Introduction

A motor block, or “freezing” event, is the sudden inability to produce effective movement, which has been documented during speech, upper extremity (UE) movements, and gait, and is often experienced by individuals with Parkinson's disease (PD) [1–4]. Freezing of gait (FOG) is arguably the most debilitating motor block, as it contributes to increased risk of falls and is associated with reduced quality of life and depression [5]. FOG is difficult to study because it is not easily elicited within the laboratory setting. Individuals with PD who experience FOG (PD+FOG) often demonstrate decreasing steplength in combination with increased cadence prior to a freezing event [4, 6]. Additionally, studies have demonstrated that people with PD+FOG exhibit greater steplength variability, increased cadence, increased step-time asymmetry, and poorer coordination compared to individuals with PD who do not experience FOG (PD-FOG) [7–9]. Plotnik et al. suggest that each of these gait parameters may have a certain level of dependency on each other, and that decline in one or more of these parameters can push an individual past the threshold for functional gait resulting in an episode of FOG [9].

Recent research investigated a possible shared mechanism between FOG and impaired upper extremity (UE) movements [10–12]. Nieuwboer et al. observed trends towards decreased coordination and increased variability of movement in freezers, nonfreezers, and controls during an alternating, high speed, and small amplitude bimanual task compared to an alternating, normal speed, and large amplitude task [11]. Additionally, they showed a strong correlation between UE freezing and Freezing of Gait Questionnaire (FOG-Q) scores. Similarly, Vercruysse et al. [10] observed UE freezing most often during alternating flexion/extension movements of the index finger during small, fast movements. Most recently, the same group [12] observed the effects of manipulating amplitude, frequency, and movement complexity (in-phase versus antiphase) during alternating flexion/extension movements of the index finger with and without auditory cueing in PD-FOG and PD+FOG. They noted that the PD+FOG group demonstrated the most movement variability during small amplitude tasks.

These results suggest that variability of UE movement and freezing of the UE (FO-UE) during bimanual tasks may be related to FOG. Additionally, FO-UE may be influenced by manipulations of amplitude and cadence that reflect characteristics of FOG, that is, small amplitude and fast cadence. However, the extent to which small amplitude or increased cadence in isolation or in combination contributes to dyscoordination of UE movement or FO-UE has yet to be determined. Further, no studies to date have compared similar manipulations of amplitude and cadence of the UE and of gait in order to gain insight into potential shared mechanisms of motor blocks in the UE and during gait.

The purpose of this study was (1) to investigate how specific manipulations of amplitude and cadence during an alternating bimanual task affect UE coordination, as measured by the phase coordination index (PCI), and number of FO-UE events and (2) to gain further insight into potential shared mechanisms between UE and gait coordination in people with PD and healthy controls. We hypothesized that decreasing amplitude or increasing cadence would decrease coordination in people with PD compared to healthy controls, with the combination of small amplitude and fast cadence eliciting the poorest coordination. Furthermore, we hypothesized that the PD+FOG group would be more affected by amplitude and cadence manipulations thereby exhibiting worse coordination and increased FO-UE episodes compared to PD-FOG and healthy controls. Finally, we hypothesized that coordination during each UE task would be correlated with coordination of a parallel gait task.

## 2. Methods

### 2.1. Participants

Twenty-eight participants with idiopathic PD (16 PD-FOG, 12 PD+FOG) and 19 healthy older adults participated. Sex, age, and disease severity characteristics are included in Table 1. Participants were recruited from the Movement Disorders Center database at Washington University in St. Louis School of Medicine (WUSM). All participants with PD had a diagnosis of idiopathic PD according to established criteria [13, 14]. Inclusion criteria included the ability to independently ambulate a minimum of twenty feet and normal or corrected to normal vision. Exclusion criteria included the presence of a diagnosed neurological or medical condition (aside from PD) and an inability to withhold anti-Parkinson medication for a limited duration. Data were collected following a minimum 12-hour overnight withdrawal of anti-Parkinson medication. Healthy older adults (>30 years old) were often the spouses of participants with PD. All healthy individuals met the above inclusion and exclusion criteria except those specific to PD. Healthy older adults were age-matched to participants with PD. Data from these individuals has been previously reported elsewhere [15]. Data were collected in the Locomotor Control Laboratory at WUSM Program in Physical Therapy. All participants gave informed consent as approved by the WUSM Human Research Protection Office. 

Participants with PD were further divided into two groups, those who experience freezing of gait (PD+FOG) and those who do not (PD-FOG), based upon a score of ≥2 on item three of the Freezing of Gait Questionnaire (FOG-Q), which indicated at least weekly freezing episodes [16]. All participants with PD participated “OFF” medication (≥12 hour withdrawal of anti-Parkinson medication). One healthy older adult was excluded from all analyses due to the inability to follow directions adequately. One participant with PD+FOG was excluded from all analyses due to inability to perform the tasks. Two additional participants with PD+FOG were excluded only from UE PCI analyses due to the inability to perform continuous alternating bilateral UE movements during one or more of the conditions. 

### 2.2. Procedure: Upper Extremity and Gait Tasks

Participants with PD were assessed by a trained research physical therapist using the Movement Disorder Society Unified Parkinson's Disease Rating Scale Motor Subscale III (MDS-UPDRS-3) to quantify disease severity [13] and completed the FOG-Q [16] to quantify frequency and severity of FOG events. All participants completed four UE tasks and four gait tasks: Baseline, Fast, Small, and SmallFast. 

A full description of the methods used during the parallel gait tasks are reported in Williams et al [15]. In short, all participants were assessed while walking at a preferred speed across a 4.9 m GAITRite instrumented walkway (CIR Systems, Inc., Sparta, NJ, USA) placed on a level surface in a large open room. For this experiment, these data were used to determine the cadence of each individual's UE task. Ten trials were performed to obtain an average baseline cadence for each individual and each trial was visually monitored for FOG events or atypical gait events such as stumbles, falls, or lateral deviation off of the GAITRite mat. Any trials consisting of these events were removed and repeated. 

During the UE tasks, participants were seated comfortably at a table in an open room. Each individual performed alternating, bilateral UE movements under four conditions: Baseline (baseline cadence, 10 cm target), Fast (+50% baseline cadence, 10 cm target), Small (baseline cadence, 5 cm target), and SmallFast (+50% baseline cadence, 5 cm target). Baseline UE cadence was determined by an individual's cadence during preferred gait as reported in Williams et al. [15]. That is, if a person walked at a rate of one step per second, we had him/her perform UE movements to one reach per second. All conditions were randomized. 

**Figure 1:**
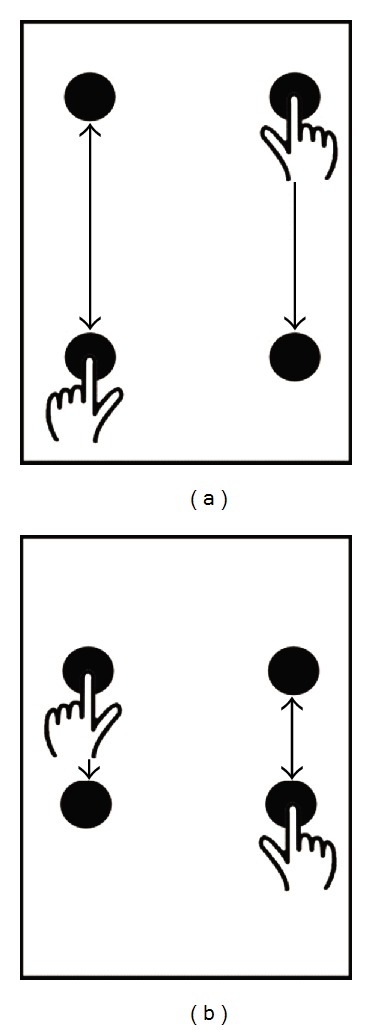
Schematic of targets and movements for the bilateral, alternating upper extremity task. Baseline (left, 10 cm); Small (right, 5 cm).

Five, 15-second trials of kinematic data for each condition were captured using 8 Hawk cameras and Cortex data acquisition software (Motion Analysis Corporation, Santa Rosa, CA, USA). Prior to each recorded trial, the participant was given a 20.32 cm × 27.94 cm (8 × 11 in) sheet marked with the appropriate targets (Figure 1). Instructions were given to use his/her index fingers to tap the targets, alternately tapping the left front/right rear targets and then the left rear/right front targets simultaneously. A metronome was turned on to the appropriate cadence while the individual tapped the targets. Once the individual practiced with the targets and metronome, the metronome was turned off and the targets were removed without the individual stopping his/her UE movement. The 15-second trial was captured after the visual and auditory cues were removed. This allowed for observation of the participant's internally generated movement state during each condition. Further, auditory and visual cues were removed as these cues are known to enhance performance in individuals with PD [14, 17, 18], and the purpose of this study was to observe each participant's internally generated movement without external cues. 

### 2.3. Outcome Variables

A quantitative assessment of freezing episodes based upon established definitions [10] and phase coordination index (PCI) were the primary outcomes. PCI was developed to quantify interlimb coordination during gait by taking into account the accuracy and consistency of the timing of stepping phases [19]. Higher PCI values indicate poorer coordination. Previous investigations have used PCI to quantify temporal coordination of steps during gait by measuring the timing of consecutive footfalls [8, 19]. In the current study, we use the same metric to assess the temporal coordination of alternating UE movements. In this case, each “footfall” in the standard PCI calculation was represented by the index finger making contact with the target furthest from the body. Therefore, only the time of taps aimed at the target furthest from the body were analyzed. A “stride” was defined as two consecutive taps of the same finger. A “step” was defined as consecutive taps of alternating fingers and from hereon will be referred to as a “cycle.” For three consecutive taps, the phase (*φ*) was determined as cycle time divided by “stride” time and scaled to 360° (*φ*_*i*_):

(1) $$\begin{array}{c} \varphi_{i}=\;360\;\times \frac{t_{Si}-\;t_{Li}}{t_{L(i+1)}-\;t_{Li}}, \end{array}$$

where *t*_*S*(*i*)_ and *t*_*L*(*i*)_ represent the timing of the *i*th finger contact of the UE with shorter and longer average “step” times, respectively. Once *φ*_*i*_ had been determined, 180 was subtracted from each *φ*_*i*_ value. The absolute value of each data point was calculated, and the mean of the array was taken to produce a measure of temporal accuracy (*φ*_ABS_):

(2) $$\begin{array}{c} \varphi_{\text{ABS}}=\bar{\left|(\varphi_{i})-\;180^{{}^{\circ}}\right|} \end{array}$$

The degree of consistency of *φ* was calculated as the coefficient of variation of *φ* values (*φ*_CV_) and given as a percentage. PCI was then calculated as

(3) $$\begin{array}{c} \text{PCI}=\varphi_{\text{CV}}+P\varphi_{\text{ABS}}, \end{array}$$

where *Pφ*_ABS_ = 100(*φ*_ABS_/180). Periods of freezing, as defined in the following paragraph, were not included in the PCI analysis. 

**Figure 2:**
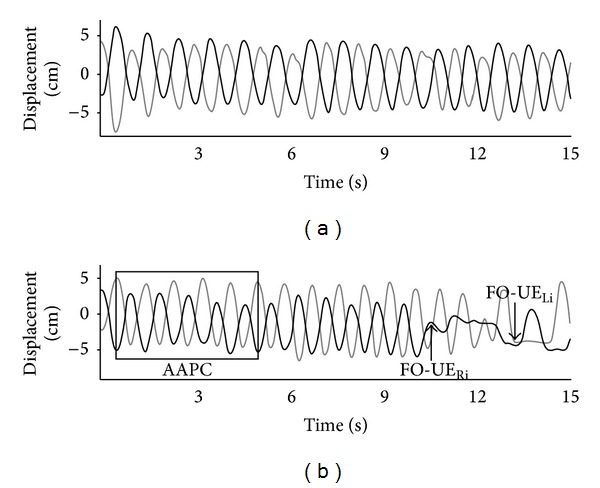
Kinematic trajectories (a) healthy older adult and (b) individual with PD-FOG with an FO-UE event. AAPC: average antiphase cycle; FO-UE_Ri_: initiation of right upper extremity freeze; FO-UE_Li_: initiation of left upper extremity freeze.

For the quantitative assessment of FO-UE episodes, trials were analyzed for the presence of FO-UE episodes by a blinded rater. In order to assess FO-UE episodes, we determined the duration and amplitude of the average antiphase cycle (AAPC) [10]. The AAPC was calculated using the first six consecutive cycles of alternating UE movement in each trial. FO-UE episodes were then defined using the calculated AAPC for a given trial. FO-UE episodes were defined as a sudden halt or decrease in amplitude of movement, which deviated from the calculated AAPC in one of two ways: (1) UE movement halted for ≥75% of the AAPC duration or (2) UE movement amplitude that was ≤50% of the AAPC amplitude, was accompanied by an irregular cycle frequency, and continued as such for at least twice the AAPC duration [10]. Additionally, voluntary stops and in-phase movements were excluded from assessment. A normal and an FO-UE event trajectory are illustrated in Figure 2.

As a secondary analysis, we determined correlations between FO-UE, UE PCI, PCI during parallel gait tasks (as reported in Williams et al. [15]), and FOG-Q score. 

### 2.4. Data Processing

Kinematic data were processed using Motion Monitor software (Innovative Sports Training, Inc., Chicago, IL, USA) and analyzed with custom written Matlab software (MathWorks, Natick, MA, USA). Position and velocity data were low pass filtered at 10 Hz before kinematic analyses. Each group's average amplitude, cadence, and PCI values for each task were determined. 

### 2.5. Statistical Approach

The same statistical approach as reported in Williams et al. [15] was used to analyze UE PCI. Mixed model repeated measures ANOVA with an unstructured covariance structure was implemented using SAS v 9.3 (SAS Institute, Inc., Cary, NC, USA). Group was used as the between subject factor and condition as the within-subject factor. We corrected for multiple comparisons by dividing *α* = 0.05 by the number of comparisons made (Bonferroni correction); a post hoc *P* value of 0.004 was considered significant for evaluating interactions. Additionally, we compared number of FO-UE episodes between PD−/+FOG groups, which was analyzed as percent of trials with FO-UE episodes. Data were rank-transformed prior to performing a repeated measures ANOVA. 

Spearman's correlation was used to determine relationships of FO-UE events with UE PCI, PCI during gait, and FOG-Q score and of UE PCI with PCI during gait as reported in Williams et al. [15]. Aside from evaluating interactions, a *P* value of ≤0.05 was considered significant for all statistical analyses.

## 3. Results

**Figure 3:**
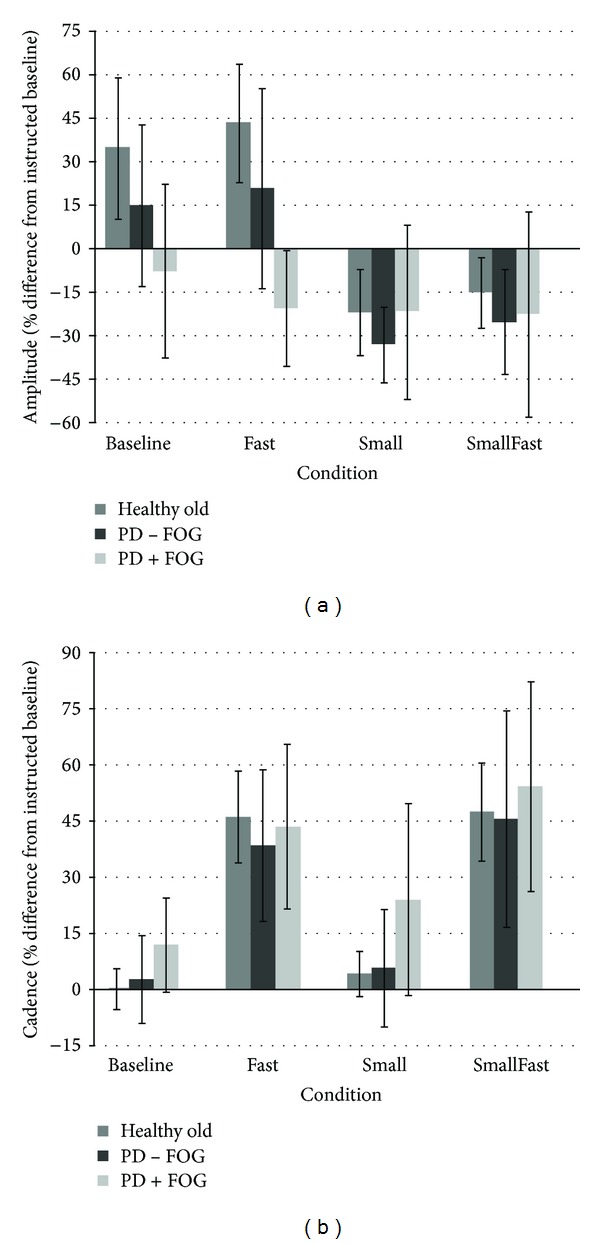
Task performance amplitude (a) expressed as percent difference from baseline (10 cm) and cadence (b) expressed as percent difference from each participant's baseline gait cadence (i.e., baseline). Bars represent standard deviations. Healthy old were different from PD−/+FOG in amplitude; there were no differences between groups in cadence.

Mean performance ± standard deviation of each group is shown in Figures 3(a) and 3(b). Values are expressed as percent difference from instructed baseline. As such, ideal performance in the Baseline condition would have cadence and amplitude values of 0%. Ideal performance in the Fast condition would have cadence values of +50% and amplitude values of 0%. Ideal performance in the Small condition would have cadence values of 0% and amplitude values of −50%. Ideal performance in the SmallFast condition would have values of +50% for cadence and −50% for amplitude. Overall, there was no between-group difference in performance of cadence (*P* = 0.21), while there was a difference between healthy older adults and individuals with PD in performance of amplitude (*P* ≤ 0.02).

### 3.1. Quantitative Assessment of FO-UE

Total numbers of FO-UE episodes for each group are reported in Table 2. There was no difference between conditions (*P* = 0.61) in percent of trials with FO-UE. A trend toward significance was present between PD-FOG and PD+FOG in percent of trials with FO-UE (*P* = 0.07). 

### 3.2. Phase Coordination Index (PCI) during Upper Extremity Tasks

**Figure 4:**
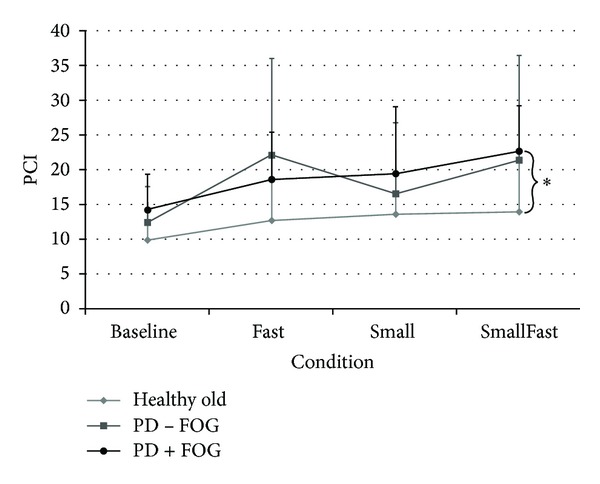
Phase coordination indices (PCI) for PD−/+ FOG and healthy older adults. *Group significantly different, *P* < 0.004.

Overall, UE PCI values were different between groups (*P* = 0.005) and conditions (*P* < 0.001), and a group by condition interaction effect was observed (*P* = 0.05) (Figure 4). Post hoc analyses showed that PD+FOG had poorer coordination compared to healthy older adults during the SmallFast condition (*P* < 0.001). 

### 3.3. Correlational Analyses

All groups were included in the analysis between UE PCI and gait PCI. Healthy older adults were excluded from analysis of FO-UE and FOG-Q, as freezing is specific to PD. UE PCI was correlated with the number of FO-UE events in the Baseline and Small conditions (Table 3). Gait PCI was correlated with UE PCI for the SmallFast (rho = 0.34; *P* = 0.03) condition. Furthermore, FOG-Q scores were correlated with FO-UE events during Fast (rho = 0.45; *P* = 0.02). FOG-Q scores were not correlated with UE PCI. Additionally, UPDRS scores were correlated with UE PCI (rho = 0.41, *P* = 0.04) but were not correlated with the number of FO-UE episodes (rho = 0.21, *P* = 0.29). 

## 4. Discussion

The results from this study demonstrate that dyscoordination and FO-UE can be elicited by manipulating cadence and amplitude of an alternating UE bimanual task. Contrary to our hypothesis, there was no difference between participants with PD and healthy controls in PCI during Small or Fast conditions. Additionally, there was no difference between PD−/+ FOG in PCI during any condition. However, PD+FOG were more affected by the combination of SmallFast, which resulted in poorer coordination in PD+FOG compared to healthy older adults. A trend toward significance between PD−/+FOG was also observed in the percent of trials exhibiting FO-UE episodes. Although periods of freezing were excluded from the PCI calculation, UE PCI and the quantitative assessment of FO-UE events were correlated during the Baseline and Fast conditions. An additional relationship was demonstrated between PCI during the SmallFast gait task and the parallel SmallFast UE task. Further, FOG-Q scores were correlated with FO-UE during the Fast condition.

Previous work demonstrated that bimanual, antiphase movement coordination is impaired in people with PD compared to healthy controls [3, 10, 12]. In keeping with this, a number of FO-UE events were elicited in this study and people with PD+FOG had poorer coordination during the SmallFast task compared to healthy controls. Further, FOG was not elicited during the parallel gait tasks reported in Williams et al. [15]. This suggests that FO-UE may be elicited more easily than FOG in individuals with PD [3, 10–12]. 

Previous work demonstrated that FO-UE was more common in more complex tasks, that is, anti-phase movement with a small amplitude and fast frequency [10], and participants with PD+FOG exhibited increased difficulty with coordination compared to participants with PD-FOG [12]. This work also suggested that FO-UE increased with small amplitude movements [10]. However, in the present study, there were no significant differences between conditions in number of FO-UE events. Only 27% of participants with PD+FOG exhibited FO-UE during the SmallFast condition, while 54% exhibited FO-UE during the Fast condition. For individuals with PD, the Small condition only accounted for 20% of the total number of FO-UE events. Additionally, there was no significant difference between the PD−/+FOG groups in the assessment of PCI and only a trend toward significance in the quantitative assessment of FO-UE episodes. In fact, two participants with PD-FOG exhibited FO-UE in each of the four conditions, and 37% of PD-FOG exhibited FO-UE during the SmallFast condition. This difference may be due to the way the participants with PD−/+FOG were qualified. 

Prior studies have qualified individuals with PD+FOG as experiencing monthly or more frequent FOG episodes [10, 12]. In the present study, we defined PD+FOG as those individuals with PD experiencing weekly or more frequent FOG episodes (score of ≥2 on item 3 of the FOG-Q). Four participants in this study reported experiencing FOG once per month (score of ≥1 on item 3 of the FOG-Q). To determine if these individuals were indeed driving the difference between our work and prior studies, we did a secondary analysis wherein the four participants with FOG once per month were placed in the PD+FOG group. Using this alternate classification scheme, we again analyzed differences in the percent of trials with FO-UE between the PD−/+FOG. This analysis yielded the same results as the original analysis; that is, there was no difference between PD−/+FOG in percent of trials with FO-UE episodes. There was also no difference between conditions of percent of trials with FO-UE events. As such, the differences in results of the present study compared to results of previous work are unlikely due to PD−/+FOG method of classification. 

It has been hypothesized that freezing may be a somatotopic phenomenon, which initially affects the UE or LE and may eventually come to impact both UE and LE tasks [10, 11]. Interestingly, two of the four participants in the PD-FOG group who reported FOG once per month accounted for 67% of FO-UE events in the Baseline and Small condition and 40% of FO-UE events in the Fast condition. Though none of the four experienced FO-UE during the SmallFast condition, those in the PD-FOG group who did may experience motor blocks of the UE and not yet experience FOG. This may also explain why not all of those with PD+FOG experienced FO-UE.

Based upon the results of the present study, it remains unclear whether FO-UE and FOG are related. However, FO-UE can be elicited by manipulating amplitude and frequency characteristics in a way that mimics changes in these variables just before an episode of FOG. The group of Nieuwboer et al. demonstrated a strong correlation between FO-UE episodes and the FOG-Q [10–12]. There may be common mechanisms underlying FO-UE and FOG, but further research is needed to investigate this, as the FOG-Q score was correlated with number of FO-UE events only during the Fast condition. Additionally, the number of FO-UE events was correlated with poor gait coordination (i.e., gait PCI) during the parallel SmallFast task, but no FOG episodes were elicited during this gait task. 

To our knowledge, this is the first time that gait coordination, that is, PCI, has been used to correlate interlimb coordination during UE tasks with gait coordination of parallel tasks. Prior work demonstrated that individuals with PD+FOG exhibit ongoing movement impairments during gait, that is, greater steplength variability and increased cadence compared to individuals with PD-FOG [7–9]. Our work supports this as participants with PD+FOG made, on average, smaller movements during the Fast condition and faster movements during the Small condition than the two other groups. 

It remains unclear whether decreased amplitude, increased cadence, or a combination of the two is associated with the freezing mechanism of the UE. Vercruysee et al. [10] conclude that smaller amplitudes elicit more FO-UE, but there were no significant differences between conditions in the present study. The differences between the present study and the previous literature suggest that small amplitude, fast cadence, or a combination of small, fast movements may not be the sole contributors to FO-UE episodes. As Plotnik et al. suggest with FOG [9], we suggest that FO-UE episodes may represent a culmination of breakdown in several aspects of control. This breakdown can be elicited by alternating bimanual Small tasks, Fast tasks, or SmallFast tasks in people with PD as measured by our quantitative assessment of FO-UE events. Though cadence and amplitude immediately prior to a FO-UE event were not measured in this study, as with FOG, we hypothesize that FO-UE is preceded by involuntary simultaneous decreasing amplitude with an accompanying hastened cadence that either a Fast, Small, or SmallFast task has the potential to elicit this response in the UE. 

Functional, complex, rhythmical tasks that require manual coordination include typing, handwriting, playing an instrument, and certain forms of exercise such as UE strength training. These tasks can replicate Small, Fast, or SmallFast conditions depending on an individual's ability. As demonstrated in the present study, decreased amplitude and increased cadence alone or together can elicit FO-UE. FO-UE during daily tasks can severely impact an individual's form of communication, hobbies, and quality of life. It is therefore important to educate patients with PD regarding these functional tasks that may elicit FO-UE.

Limitations of this study are acknowledged. First, only one independent rater determined the presence of FO-UE based upon established definitions [10], and reliability of this method was not established. Further, preselected amplitudes and cadence were utilized and we cannot say whether a large amplitude or slow cadence would have elicited the same or lesser amount of dyscoordination or FO-UE. Additionally, cadence was determined from a gait task rather than from an UE movement task. This methodology was employed as the gait task provided a parallel motor task, without introducing the UE task and allowing for motor learning effects to bias the study. We acknowledge the difference between UE and lower extremity tasks and that perhaps movement frequency may be a higher in UE tasks. Additionally, participants were not sex-matched, participants with PD were not matched for disease severity, and UPDRS scores were correlated with PCI. We cannot conclude definitively whether our measures of dyscoordination or FO-UE are due to disease severity, FOG status, or both. Finally, the sample size of this study was relatively small with large amounts of variation within each condition per group, which makes it difficult to detect significant differences between groups and conditions. 

## 5. Conclusions and Future Direction

Imposed manipulations of cadence and amplitude that mimic changes in gait associated with FOG can affect UE coordination and elicit FO-UE episodes in people with PD. People with PD+FOG have poorer coordination compared to healthy controls during a SmallFast task, but no other differences in UE coordination were noted between healthy controls and individuals with PD. FO-UE and FOG may be related, but future research is needed to explore potential links between the two. Future clinical studies could also examine the utility of instructions to increase movement amplitude and decrease movement cadence as a means of enhancing coordination and reducing FO-UE and FOG.

## Figures and Tables

**Table 1:** Final sample characteristics.

Characteristic	Healthy old *N* = 18	PD-FOG *N* = 16	PD + FOG *N* = 11
Sex (M/F)^∗†^	6/12	5/11	10/1
Age (yrs)^‡^	68.4 ± 7.5	67.6 ± 9.5	70.8 ± 6.9
Average baseline amplitude (cm)*	13.5 ± 2.4	11.4 ± 2.8	9.2 ± 2.9
Average baseline cadence (taps per minute)	114.4 ± 10.5	156.1 ± 23.9	151.71 ± 31.5
Hoehn and Yahr OFF^¥^		2.2 ± 0.44	2.2 ± 0.26
MDS-UPDRS-3 OFF^∗§^		26.1 ± 9.4	44.8 ± 11.8
FOG-Q score^∗¥^		2.8 ± 1.8	11.3 ± 2.2

*All group(s) significantly different; *P* < 0.05.

^†^Chi-square analysis; ^‡^one-way ANOVA; ^*¥*^Mann-Whitney *U* Test; ^§^Independent samples *t*-test.

Abbreviations:

M: male.

F: female.

Yrs: years.

MDS-UPDRS-3: Movement Disorder Society Unified Parkinson Disease Rating Scale Motor Subscale 3.

FOG-Q: Freezing of Gait Questionnaire.

**Table 2:** Number of FO-UE episodes.

Condition	Group		
	Healthy old (*n* = 18)	PD-FOG (*n* = 16)	PD + FOG (*n* = 11)
Baseline			
FO-UE	0 (0%)	4 (3.7%)	10 (11.0%)
*N*	0	2	4
Fast			
FO-UE	0 (0%)	5 (6.2%)	12 (21.8%)
*N*	0	4	6
Small			
FO-UE	0 (0%)	4 (3.7%)	8 (14.5%)
*N*	2	5
SmallFast			
FO-UE	1 (0.01%)	12 (11.2%)	6 (9.5%)
*N*	1	6	3

Values are number of episodes of freezing per condition (percent of total trials with at least one episode in parentheses). *N*: total number of individuals experiencing ≥1 episode of FO-UE.

Abbreviations:

FO-UE: freezing of the upper extremity.

PD-FOG: Parkinson disease without freezing of gait.

PD + FOG: Parkinson disease with freezing of gait.

**Table 3:** Spearman's correlations between UE PCI, Gait PCI, FOG-Q, and number of FO-UE events.

	FO-UE	UE PCI
Baseline		
UE PCI	0.41*	—
Gait PCI	−0.22	0.18
FOG-Q	0.27	0.09
Fast		
UE PCI	0.10	—
Gait PCI	0.04	0.15
FOG-Q	0.45*	−0.05
Small		
UE PCI	0.66**	—
Gait PCI	0.28	0.19
FOG-Q	0.30	0.27
SmallFast		
UE PCI	−0.32	—
Gait PCI	0.06	0.34*
FOG-Q	−0.12	0.21

*P < 0.05.

**P < 0.01.

Abbreviations:

UE: upper extremity.

PCI: phase coordination index.

FOG-Q: freezing of gait questionnaire.

FO-UE: freezing of upper extremity.

## References

[1] Fahn S (1995). The freezing phenomenon in parkinsonism. *Advances in Neurology*.

[2] Moreau C, Ozsancak C, Blatt JL, Derambure P, Destee A, Defebvre L (2007). Oral festination in Parkinson’s disease: biomechanical analysis and correlation with festination and freezing of gait. *Movement Disorders*.

[3] Almeida QJ, Wishart LR, Lee TD (2002). Bimanual coordination deficits with Parkinson’s disease: the influence of movement speed and external cueing. *Movement Disorders*.

[4] Giladi N, Nieuwboer A (2008). Understanding and treating freezing of gait in Parkinsonism, proposed working definition, and setting the stage. *Movement Disorders*.

[5] Bloem BR, Hausdorff JM, Visser JE, Giladi N (2004). Falls and freezing of gait in Parkinson’s disease: a review of two interconnected, episodic phenomena. *Movement Disorders*.

[6] Nieuwboer A, Dom R, de Weerdt W, Desloovere K, Fieuws S, Broens-Kaucsik E (2001). Abnormalities of the spatiotemporal characteristics of gait at the onset of freezing in Parkinson’s disease. *Movement Disorders*.

[7] Moreau C, Defebvre L, Bleuse S (2008). Externally provoked freezing of gait in open runways in advanced Parkinson’s disease results from motor and mental collapse. *Journal of Neural Transmission*.

[8] Plotnik M, Giladi N, Hausdorff JM (2008). Bilateral coordination of walking and freezing of gait in Parkinson’s disease. *European Journal of Neuroscience*.

[9] Plotnik M, Giladi N, Hausdorff JM (2012). Is freezing of gait in Parkinson’s disease a result of multiple gait impairments? Implications for treatment. *Parkinson’s Disease*.

[10] Vercruysse S, Spildooren J, Heremans E (2012). Freezing in Parkinson’s disease: a spatiotemporal motor disorder beyond gait. *Movement Disorders*.

[11] Nieuwboer A, Vercruysse S, Feys P, Levin O, Spildooren J, Swinnen S (2009). Upper limb movement interruptions are correlated to freezing of gait in Parkinson’s disease. *European Journal of Neuroscience*.

[12] Vercruysse S, Spildooren J, Heremans E (2012). Abnormalities and cue dependence of rhythmical upper-limb movements in Parkinson patients with freezing of gait. *Neurorehabilitation and Neural Repair*.

[13] Goetz CG, Tilley BC, Shaftman SR (2008). Movement disorder society-sponsored revision of the unified Parkinson’s disease rating scale (MDS-UPDRS): scale presentation and clinimetric testing results. *Movement Disorders*.

[14] Bryant MS, Rintala DH, Lai EC, Protas EJ (2009). An evaluation of self-administration of auditory cueing to improve gait in people with Parkinson’s disease. *Clinical Rehabilitation*.

[15] Williams AJ, Peterson DS, Earhart GM (2013). Gait coordination in Parkinson disease: effects of step length and cadence manipulations. *Gait Posture*.

[16] Giladi N, Tal J, Azulay T (2009). Validation of the freezing of gait questionnaire in patients with Parkinson’s disease. *Movement Disorders*.

[17] Rochester L, Hetherington V, Jones D (2005). The effect of external rhythmic cues (auditory and visual) on walking during a functional task in homes of people with Parkinson’s disease. *Archives of Physical Medicine and Rehabilitation*.

[18] Lee SJ, Yoo JY, Ryu JS, Park HK, Chung SJ (2012). The effects of visual and auditory cues on freezing of gait in patients with parkinson disease. *The American Journal of Physical Medicine and Rehabilitation*.

[19] Plotnik M, Giladi N, Hausdorff JM (2007). A new measure for quantifying the bilateral coordination of human gait: effects of aging and Parkinson’s disease. *Experimental Brain Research*.

